# Conversion of primary liver cancer after targeted therapy for liver cancer combined with AFP-targeted CAR T-cell therapy: a case report

**DOI:** 10.3389/fimmu.2023.1180001

**Published:** 2023-05-15

**Authors:** Yun Wang, Yan Zhao, Miaoling Li, Huilian Hou, Zhijie Jian, Weizhi Li, Peijie Li, Fuquan Ma, Mengying Liu, Haibo Liu, Hui Xue

**Affiliations:** Department of Gastroenterology, The First Affiliated Hospital of Xi’an Jiaotong University, Xi’an, Shaanxi, China

**Keywords:** primary liver cancer (PLC), hepatocellular carcinoma (HCC), chimeric antigen receptor T cell (CAR-T), AFP, immunotherapy, case report

## Abstract

Primary liver cancer (PLC) that originates in the liver is a malignant tumor with the worst prognosis. Hepatocellular carcinoma (HCC) is the most common type of PLC. Most PLC cases are diagnosed at advanced stages mainly due to their insidious onset and rapid progression. Patients with PLC undergo surgical intervention or localized treatment, but their survival is often affected by its high relapse rate. Medical treatment is the primary option for patients with liver cancer, especially with advanced extrahepatic metastases. Molecular targeted therapy exerts an anti-tumor effect by acting on various signaling pathways involved in molecular pathogenesis; however, high drug resistance and low therapeutic responsiveness of PLC to molecular targets challenge the treatment option. In recent years, after surgical intervention, radiotherapy, chemotherapy, and/or molecular targeted therapy, autologous cell immunotherapy has been adopted for PLC. As a typical autologous cell immunotherapy, CAR T-cell therapy uses genetically modified T cells to express tumor-specific chimeric antigen receptors (CARs). Its targeting ability, persistent nature, and tumor-killing function result in a significant impact on the treatment of hematological tumors. However, no breakthrough has happened in the research specific to the curation of lung cancer, liver cancer, breast cancer, and other common solid tumors. In this context, a combination of molecular targeted therapy and CAR T-cell therapy was used to treat a patient with advanced HCC to achieve a partial remission(PR) and facilitate further liver transplantation.

## 1 Introduction

Primary liver cancer (PLC) is the most common malignant tumor and can be divided into hepatocellular carcinoma (HCC), cholangiocarcinoma, and combined hepatocellular and cholangiocarcinoma by histological type (1). HCC accounts for over 80% of cases of PLC (2). Clinicians always look for innovative therapy for PLC since it carries the most risk and worst prognosis. From the latest statistical data, about 600,000 new cases of liver cancer occur every year, and liver cancer is the 6th most common cancer and the 4th leading cause of cancer death worldwide (3). Further, it is a top killer cancer in China due to its high morbidity, high mortality, and poor prognosis (4).

PLC is always under discussion for basic research since it has the standard characteristics of a solid tumor. As medical technologies advance continuously, besides traditional tumor resection and liver transplantation, new first-line methods, such as transarterial chemoembolization (TACE), transarterial radioembolization (TARE), percutaneous radiofrequency ablation (RFA), external chemoradiation is used in patients with early stages of PLC or poor liver function, to reduce the tumor size to enable complete resection of tumors (5, 6). A combination treatment of TACE and RFA for patients with HCC showed a better curative effect than a single application (7).

A combination of molecular targeted and immune-based therapy has gradually become the primary treatment strategy for advanced liver cancer to improve the survival of patients (8). The diagnostic and treatment norms of PLC have been continuously updated using patient-centered, multidisciplinary comprehensive approaches with actual cases as templates to benefit more patients. With the evolution of tumor molecular biology and the emergence of precision medicine, the treatment of liver cancer is more individualized.

New-generational anti-tumor drugs have been investigated in treating advanced liver cancer due to their specificity and targeting ability. Since HCC is a multifocal hemangioma and angiogenesis mediates tumor progression, a representative therapy targets the epidermal growth factor receptor (EGFR) and the vascular endothelial growth factor (VEGF). The EGFR has tyrosine kinase activity, participating in cellular physiological processes, closely related to the proliferation, invasion, and metastasis of tumor cells. The EGFR is over-expressed in various solid tumors. Clinical studies confirmed that tyrosine kinase inhibitors such as sorafenib inhibited tumor angiogenesis by intervening in the RAF/MEK/ERK pathway, and hindered the proliferation and survival of tumor cells via RAF kinase signaling-dependent and signaling-independent mechanisms (9), thereby extending the mean overall survival of patients with advanced HCC by nearly 3 months (10). Besides sorafenib, regorafenib, a multi-kinase inhibitor targeting VEGFR1-3, c-kit, RET, BRAF, PDGFR, and FGFR, demonstrated acceptable tolerability and anti-tumor activity in patients with intermediate or advanced HCC as second-line therapy after first-line treatment with sorafenib (11). Overall, molecular targeted therapy can precisely eradicate tumors, but its drug resistance is unavoidable due to the genetic mutations on the targeted sites of cancer cells; however, its efficacy can be built with multiple treatment cycles.

Immunotherapy subsequently emerged and became the fourth most important treatment for cancer after surgery, chemotherapy, and radiation. The most recent systemic anticancer therapy, divided into active immunotherapy and adoptive immunotherapy, is cancer immunotherapy, which, as the name implies, treats cancer by activating the immune system. Active immunotherapy stimulates or amplifies anticancer immune responses in cancer patients, such as the use of cancer cell vaccines to boost immune responses. The other most common example is CAR-T therapy, which was used in the patient in this case, is to isolate immunocompetent cells from the peripheral blood of the patient, genetically modify them, expand them *in vitro*, and functionally characterize them, and then infuse them back into the patient, to achieve a kind of adoptive immunotherapy (ACT) that directly kills the tumor or indirectly kills the tumor cells by stimulating the body’s immune response (12, 13). Nowadays, enhanced CAR T-cell therapy has been used as an effective alternative treatment of various hematological tumors, It has made great progress in hematologic malignancies. To date, the FDA has approved five CAR T therapies, such as CD19-specific CAR T cells for lymphoblastic leukemia and CAR T cells targeting B cell maturation antigen (BCMA) for multiple myeloma (14). But it still faces more challenges in the treatment of solid tumors due to their antigen heterogeneity, the immunosuppressive tumor microenvironment (TME), as well as the inaccessibility of drugs to tumors (15). Currently, a number of studies have focused on a few malignant solid tumors, including Biliary tract cancer (16), gastrointestinal tumors (17), Glioblastoma (18), nonsmall-cell lung cancer (19), Ovarian Carcinoma (20), and HCC (21), and the targeted molecules of CAR include EGFR, CD133, HER-2/neu, FRα, GPC3, etc, although many of them were terminated due to lack of objective clinical response or safety concerns.

In summary, CARs are a new class of immunotherapy with antigen presentation, targeting the ability to overcome immune escape, and have considerable potential in the treatment of cancer. The most significant advantages of CARs include non-MHC identification and killing of tumor cells, significantly increasing the survival rate of patients. Compared with chemical drugs and biological agents, CAR T cells can last longer in patients to improve immune tolerance. Moreover, as mentioned above, some recent studies and clinical trials found that CAR T-cell therapy targeting GPC was relatively safe and effective in patients with HCC, requiring further exploration (22, 23). In addition, Liu et al. (24)designed and developed a CAR T cell for targeting the AFP peptide-MHC complex against liver cancer. It’s just the molecule that was targeted for therapy in this case.

Since the immunosuppressive TME in solid tumors and the physical characteristics of tumor tissues can hinder the efficacy of CAR T-cell therapy, the combined use of CAR T-cell therapy with targeted drugs that can regulate the TME can promote the anti-tumor effect (25–27). A previous study showed that the combined use of cyclophosphamide and CEA-specific CAR T cells increased the cytotoxic activity of anti-CEA CAR T cells (28). Furthermore, another study with the GPC-3-specific CAR T cells in combination with sorafenib in immunodeficient mice eradicated their large tumor xenografts (29) and facilitated further clinical research. The next part of this paper describes a case with PLC, treated with targeted drugs and AFP-targeted CAR T cells, showing significant benefits from the combination therapy. Additionally, this paper presents all identifiable images and data of the patient on the basis of written informed consent from the patient.

## 2 Case report

A 52-year-old male patient was admitted to the Department of Gastroenterology of the First Affiliated Hospital of Xi’an Jiaotong University in December 2017, with “intermittent abdominal pain in the right upper quadrant for one month, which was aggravated for the past 2 days”. He had a 30-year history of positive Hepatitis B surface antigen (HBsAg) without any treatment, a four-year history of hypertension, and a history of drinking alcohol over 10 years. On clinical examination, he showed stable vital signs, an appearance suggesting chronic liver disease, palmar erythema, no obvious abnormalities from the cardiopulmonary examination, abdominal distension, visible epigastric veins on the upper abdominal wall, an umbilical hernia, positive abdominal tenderness on the right upper quadrant without rebound pain, undesired results of liver and spleen palpation, no shifting dullness, no obvious abnormalities on the spine and extremities, and no abnormalities on the neurological examination. Upon medical imaging examination on December 20, 2017, chest and upper abdominal CT showed multiple micro-nodules on both lungs, suggesting metastases and multiple low-density shadows on the liver accompanied by liver shrinkage, warranted further examination, while abdominal DCE MRI revealed multiple enhanced nodular foci on the liver. Furthermore, a gastroscopy on December 22, 2017, showed mild to moderate varicosity of esophageal veins and the gastric fundus veins, and the serum biochemical profile showed a hepatic function of Child-Pugh class B, an AFP level of 21,223 ƞg/mL, and an HBV-DNA level of 6.5E+6 IU/mL. Moreover, PET-CT on December 26, 2017, showed heterogeneous increased radionuclide uptakes in multiple regions of the liver and the lungs. Based on all examination results, he was diagnosed with PLC with bilateral lung metastases, hepatitis B-related cirrhosis, liver decompensation, (Barcelona Clinic Liver Cancer) BCLC stage C, and an (Eastern Cooperative Oncology Group) ECOG score of 1.

Following admission, he was given symptomatic and supportive treatments, such as anti-hepatitis B virus treatment, liver protectives, drugs to improve blood coagulation, and supplementation for hematopoiesis, as well as sorafenib therapy following regorafenib targeted therapy and CAR T-cell immunotherapy. From December 22, 2017, the anti-virus combination therapy of Entecavir and Tenofovir was used. When the re-examination of the virus showed negative on April 28, 2018, only Tenofovir anti-virus therapy has been used since then. Specific to the anti-tumor treatment, targeted drugs were administered before the CAR T-cell therapy. Sorafenib targeted therapy was used from December 29, 2017, which was successively replaced by regorafenib targeted therapy at 160 mg from January 19, 2018, 120 mg from April 20, 2018, 80 mg from May 20, 2018, no targeted drugs from November 7, 2018, and 80/120 mg alternatively between February 18, 2019, and December 30, 2019. From 27 January 2018, the targeted therapy was combined with 23 times re-infusion of CAR T cells (once every 2-4 weeks). The total treatment lasted for 23 months.

CAR-T cell preparation involves extracting 60-100ml of peripheral venous blood from patients 2 to 3 weeks prior to treatment, isolation and purification of T cells by cell washers *in vitro*, and removal of impurities such as granulocytes, red blood cells, and anticoagulants that impact on the subsequent production of cells. Cell processing uses a closed, automated system. After activation of T cells, CAR genes are transferred using a lentiviral vector system to expand CAR-T cells *in vitro*, which are then frozen for clinical treatment. T cells carry human-derived TCR-like antibodies targeting AFP peptide/MHC complexes on the surface of hepatoma cells that selectively bind to proteasome-degraded AFP peptide fragments of tumor cells that are presented to the cell surface by major histocompatibility complexes (MHC). When antibodies bind to this complex, T cells can be activated and kill tumor cells.

**Figure 1:**
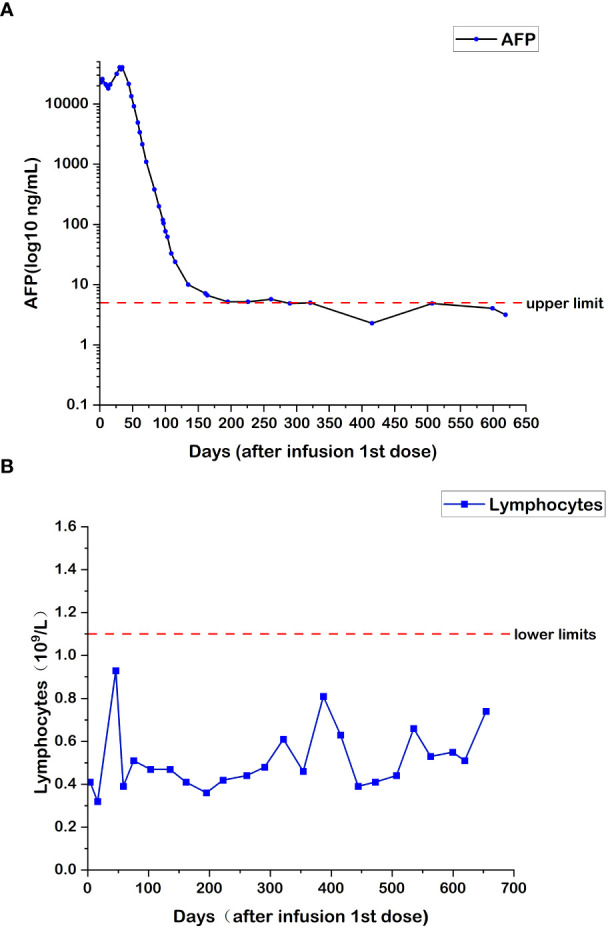
**(A)** Changes in AFP levels during CAR T-cell therapy. **(B)** Changes in lymphocyte numbers during CAR T-cell therapy.

During the treatment with combined targeted therapy and individualized cellular immunotherapy, the HCC-specific tumor marker (AFP) was determined multiple times, with a peak value of 40,451 ƞg/mL. The variations in AFP levels are shown in **Figure 1A**. Since July 2, 2018, the AFP level returned to normal during all follow-up rechecks. Meanwhile, the number of lymphocytes increased. The number of lymphocytes at the end of each cycle of CAR T-cell therapy is shown in **Figure 1B**. The amount of re-infused T cells in each cycle was 6.0×10^6^ CAR^+^ cell number/kg body weight, and the total amount was 522×10^6^/80 mL.

Chest and abdominal enhanced CT (CAECT) before the combination therapy showed irregular mass shadows in the superior segment of the right anterior lobe and multiple nodules in both lungs. Regular PET/CT in September 2018, after 8 months of treatment revealed shrinkage of lesions in the superior segment of the right posterior lobe, and the disappearance of most of the lung nodules. The CAECT on December 10, 2018, after 11 months of treatment showed that the foci on the right lobe were slightly enlarged, the metastases in both lungs disappeared, the primary foci on the liver were partially liquefied and necrotized, and no new foci were found. The CAECT after one year of treatment showed insignificant changes in the size of the hepatic lesions but increased necrosis and cystic degeneration.

**Figure 2:**
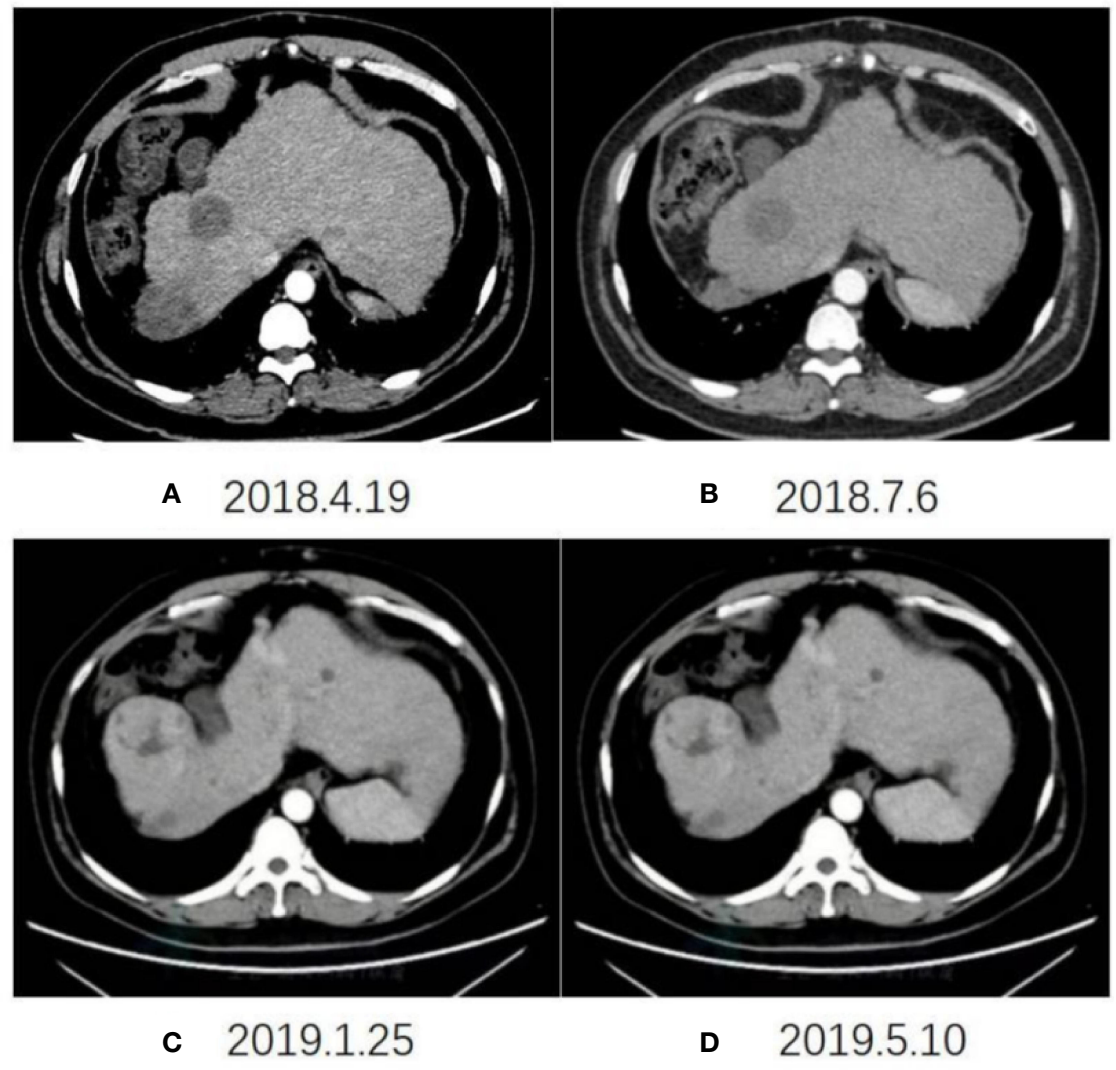
Changes in abdominal ECT after the initial CAR T-cell therapy on April 19, 2018 **(A)**, July 6, 2018 **(B)**, January 25, 2019 **(C)**, May 10, 2019 **(D)**, respectively.

**Figure 3:**
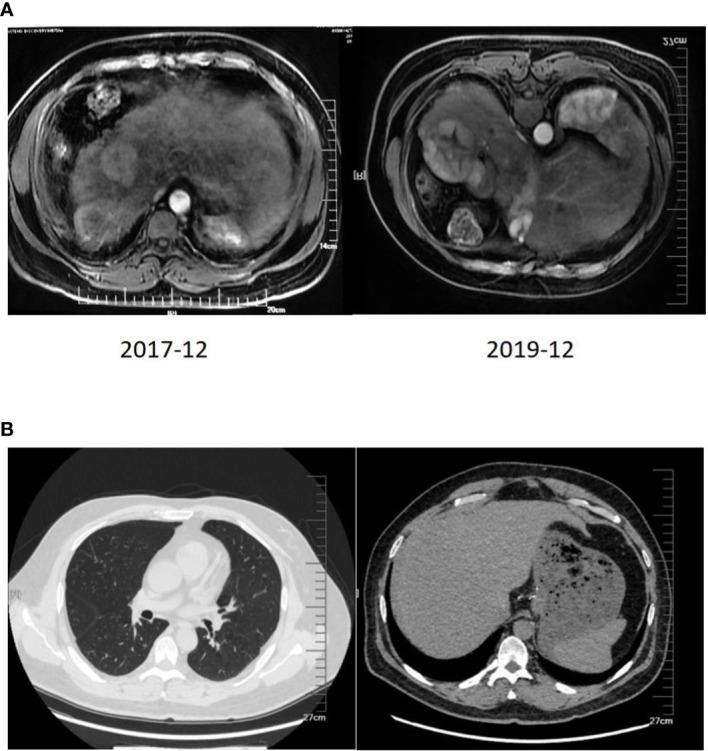
**(A)** Changes between the prior-treatment and post-treatment MRI pictures of the abdomen. **(B)** CT scan of the chest and abdomen one year after liver transplantation.

The imaging examination (abdominal ECT) results of the patient are shown in **Figure 2**, and the foci size in the liver during the treatment and at different post-treatment time points by the abdominal ECT are shown in **Table 1**, while the changes between the prior-treatment and post-treatment MRI pictures of abdomen are shown in **Figure 3A**.

**Table 1:** The changes in foci size in the liver at different time points during and after treatment by abdominal ECT.

DATE	Right anterior lobe	Right posterior lobe
2018-4	34mm	41mm
2018-7	46mm	37mm
2018-12	56mm	33mm
2019-1	66mm	30mm
2019-5	70mm	26mm

Chest ECT after one year of treatment showed no noticeable changes in the number and range of the lung nodules, and chest ECT after another 5 months with the same therapeutic scheme showed the disappearance of lung nodules. The final re-infusion of CAR T cells was completed in October 2019. The follow-up PET-CT on December 3, 2019, showed no significant changes except for the expansion of nodular necrosis in the superior segments of the right lobe and the metabolic activities in some regions.

The two-year combination therapy lowered the BCLC from stage C to stage B, reduced and maintained the AFP to a normal level, obtained a Child-Pugh class A of liver function, made most lung nodules disappear and the most hepatic foci necrotize and cystic degenerate, and obtained a PR. At this time of the treatment, whether the patient should continue to live with the tumor or undergo further conversion therapy should be decided. Based on the multidisciplinary team discussion, the patient was eligible for liver transplantation, and he underwent piggyback liver transplantation under general anesthesia on January 16, 2020, three months after the final re-infusion of CAR T cells. The surgical procedure went smoothly. The patient took immunosuppressive drugs, an anti-viral drug (Tenofovir), and anticoagulant drugs orally for a long period after surgery. Also, he continued with regorafenib at 80 mg for 3 months. The specimen of the total hepatectomy during the transplantation was submitted for examination. Pathological examination showed a giant type of moderately differentiated hepatocellular carcinoma with extensive necrosis and local cystic degeneration in the right posterior lobe, and a small type of moderately differentiated hepatocellular carcinoma with focal necrosis in the right anterior lobe. Cancer cells were arranged in a trabecular and cord-like manner according to the hematoxylin-eosin (HE) staining. Immunohistochemical staining showed GPC3(+), Hep-1(+), HSP-70(+), CK19 (–), Ki67(+20%), HBsAs (+), HBcAg (–), CD34(+), and Masson staining showed the formation of collagen fiber septa in the liver tissue, including pseudolobulations. The follow-up rechecks of the AFP level and the chest and abdominal CT (**Figure 3B**) were conducted after one year of the transplantation. The patient has now returned to normal work and life.

**Figure 4:**
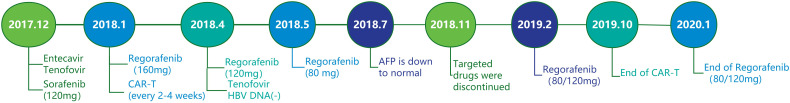
The treatment schedule for this patient.

To visually reflect the prognosis and clinical efficacy of the patients, we listed the whole treatment process and changes of important laboratory indices till surgery in **Figure 4**.

## 5 Discussion

Regorafenib is a targeted new-generational potent multi-kinase inhibitor and is used as a standard second-line treatment in patients with PLC based on current recommendations (11, 30, 31). Regorafenib not only possesses the function of anti-angiogenesis and anti-tumor proliferation, similar to the first-line therapy sorafenib, but also reduces immunosuppression, macrophage infiltration, and tumor metastasis, and targets more sites than sorafenib (32). After evaluation of tumor progression following one month of first-line therapy sorafenib, the second-line therapy was immediately started. Meanwhile, the FDA’s approval of nivolumab for patients with HCC after sorafenib therapy distinguished the era of immunotherapy for liver cancer (33). Considering the effectiveness of multifaceted combination therapy for PLC and the basis of the clinical research on T-cell immunotherapy in solid tumors from our hospital, we combined the targeted therapy with T-cell immunotherapy for this patient. Our clinical research on T-cell immunotherapy for hematological tumors achieved a breakthrough. T cells are extracted from the patients for cultivating the CAR T cells. The cultivated T cells are re-infused into the patients, and they proliferate and kill tumor cells with specific antigenicity in various cancers. Its efficacy in malignant hematological tumors has been validated, and its clinical response rate for acute lymphoblastic leukemia (ALL) has reached 90% (34).

However, in solid tumors, CAR-T remains limited in its targeting and entry of CAR-T cells into tumor tissues. AFP is expressed in 60% -80% of HCC cases, making it an ideal biomarker for evaluating CAR-mediated T cells therapy in hepatic solid tumors. However, AFP is only expressed and secreted intracellularly and cannot be targeted by conventional CARs. Because MHC I on the surface of tumor cells can process and deliver intracellular proteins, researchers designed antibodies against the AFP-MHC-I complex and built CAR, which is how CART was constructed in this case. Studies have shown that CAR-T therapy targeting intracellular antigens or secretory antigens has good antitumor effects. In addition, several studies have combined CAR-T cells with differentially targeted kinase inhibitors to find the best partner for CAR-T to improve drug efficacy. In HCC, due to the high expression of Glypican-3 (GPC3) in HCC tissues, a large number of studies have focused on GPC3-CAR-T treatment. and Wu et al. combined GPC3-CAR-T with sorafenib for HCC, and preclinical data showed that sorafenib enhanced the antitumor efficacy of GPC3-CAR-T in a mouse model (29). Based on these studies, we found that in this case, patients could achieve PR with targeted therapy combined with AFP-CAR-T cell treatment.

Specific to challenges in applying CAR T-cell therapy in the treatment of solid tumors, except for the potent efficacy for leukemia, the following challenges have been encountered. First, CAR T-cell therapy is a customized or individualized therapy in nature, and the mass production of CAR T cells is challenging. Second, the local immunosuppression in the TME of solid tumors prevents the cell number from reaching the therapeutic dosage. Although it is theoretically believed that T cells can self-proliferate, this function can only occur after binding to antigens. Third, the re-infused T cells with CARs may be regarded as foreign bodies and eliminated by the existing immune cells due to the addition of CARs since the antibodies in the CAR T cells mostly come from external sources, mostly from mice. Fourth, how to accurately deliver the CAR T cells to the tumor regions remains questionable (35–38).

In conclusion, the patient in this case scenario was fortunate. It has been 4 years since the diagnosis of PLC and now he has returned to normal life after the success of the conversion therapy. Reviewing the therapeutic strategies in this case, which therapy contributes more to the recovery or whether the combination therapy is more effective remains unclear. But the synergy between the two therapies is unquestionable. Nevertheless, this case provides a good example of using CAR T-cell therapy in treating solid tumors. Despite the multiple challenges, the case can serve as a basis for solving the difficulties in the current application of CAR T-cell therapy when combined with other therapeutic methods.

## Data availability statement

The original contributions presented in the study are included in the article/supplementary material. Further inquiries can be directed to the corresponding author.

## Ethics statement

The studies involving human participants were reviewed and approved by The First Affiliated Hospital of Xi’an Jiaotong University, Xi’an, Shaanxi, China. The patient provided his written informed consent to participate in this study. Written informed consent was obtained from the individual(s) for the publication of any potentially identifiable images or data included in this article.

## Author contributions

YW, W-ZL, P-JL, and M-YL provided patient information. YW, YZ, M-LL, and H-LH were involved in the interpretation of the data, YZ collected and organize the data, measured the size of the lesion on imaging and draw diagrams. YW, YZ, and M-LL reviewed the pathological sections. YZ, M-LL, and Z-JJ were involved in the drafting of the manuscript and analyzed the data. YW, YZ, and F-QM participated in the revisions. All authors contributed to the article and approved the submitted version.
